# Lithium in the Natural Waters of the South East of Ireland

**DOI:** 10.3390/ijerph14060561

**Published:** 2017-05-26

**Authors:** Laurence Kavanagh, Jerome Keohane, John Cleary, Guiomar Garcia Cabellos, Andrew Lloyd

**Affiliations:** EnviroCORE, Department of Science and Health, IT Carlow, Kilkenny road, Co., Carlow R93V960, Ireland; Ger.Keohane@itcarlow.ie (J.K.); John.Cleary@itcarlow.ie (J.C.); Guiomar.Garcia-Cabellos@itcarlow.ie (G.G.C.); Andrew.Lloyd@itcarlow.ie (A.L.)

**Keywords:** lithium, groundwater, surface-water, mining

## Abstract

The South East of Ireland (County Carlow) contains a deposit of the valuable lithium-bearing mineral spodumene (LiAl(SiO_3_)_2_). This resource has recently attracted interest and abstractive mining in the area is a possibility for the future. The open cast mining of this resource could represent a potential hazard in the form of metalliferous pollution to local water. The population of County Carlow is just under 60,000. The local authority reports that approximately 75.7% of the population’s publicly supplied drinking water is abstracted from surface water and 11.6% from groundwater. In total, 12.7% of the population abstract their water from private groundwater wells. Any potential entry of extraneous metals into the area’s natural waters will have implications for people in county Carlow. It is the goal of this paper to establish background concentrations of lithium and other metals in the natural waters prior to any mining activity. Our sampling protocol totaled 115 sites along five sampling transects, sampled through 2015. From this dataset, we report a background concentration of dissolved lithium in the natural waters of County Carlow, surface water at $\bar{x}$ = 0.02, SD = 0.02 ranging from 0 to 0.091 mg/L and groundwater at $\bar{x}$ = 0.023, SD = 0.02 mg/L ranging from 0 to 0.097 mg/L.

## 1. Introduction

Lithium is an uncommon parameter for drinking water analysis. Its spatial and temporal distributions are not commonly studied and are often overlooked because it appears at very low levels. Its concentrations in natural waters vary depending on geology, topography, hydrogeology and other variables. Despite this, there are some generally referred to ranges of lithium concentrations in waters. In sea water, where it is the fourteenth most abundant element, it occurs at 0.14 to 0.20 mg/L [1,2,3] and in fresh waters it occurs at 0.001 to 0.020 mg/L [3,4,5,6,7,8,9,10,11]. Shand et al. report medium levels of lithium in groundwater, associated with different bedrock geologies in England: Chalk at 0.0008 mg/L and Permo-Triassic Sandstone at 0.001 mg/L [12]. There is no legislative requirement for lithium to be monitored in Irish drinking water, therefore it has no safety standard. This does not mean that concentrations cannot reach toxic levels. The high altitude continental brine aquifers of Argentina, Chile and Bolivia contain most of the world’s accessible lithium; Bolivia alone is said to have jurisdiction over a third of the world’s recoverable lithium [13]. The lithium levels in the natural waters in these areas are very high: ≥5 mg/L. Northern Chile has recorded the highest levels of lithium in surface waters in the world, at levels of 2 to 20 mg/L, 2–3 orders of magnitude higher than most rivers. Because of this, there has been some work in the area concerning the high exposure of lithium in the population [11,14,15,16].

Lithium has recently come into public awareness because of its use in lithium-ion batteries (LIBs). LIBs are already powering most of the electronic devices we use every day, such as our phones and laptops, a market that did not exist 20 years ago. The fact that lithium is set to power the electric vehicles of the future makes it a key resource for both the industrialized world and emerging economies. Other uses of lithium include as lithium carbonate, a medication to treat mental disorders; as lithium stearate, an all-purpose high temperature lubricant; as a fluxing agent in the ceramics industry; as a light-weight alloy; and as lithium chloride, a hygroscopic agent in air conditioning systems. The emerging electric vehicle market is pushing this ordinary alkali metal to become the new “white petroleum” of the 21st century. It is the objective of this work to establish a background level of lithium in the waters of Carlow, prior to any future commercial abstraction.

Lithium has been used as a mood stabilizing drug in people with mood disorders such as bipolar and major depression for over 150 years. It has also been used successfully to treat people with suicidal tendencies. There are several studies which suggest a negative association between lithium levels in drinking water and suicide rates in populations [17,18,19,20]. Large doses of lithium (10 mg/L in serum) are used in patients to treat these disorders. Lithium has a narrow therapeutic index: at 10 mg/L of blood, a person is considered mildly lithium poisoned; at 15 mg/L, they experience confusion and speech impairment; at 20 mg/L, there is a risk of death [11]. The lithium ion displays chronic toxicity on the human central nervous system [11,21,22]. A lethal dose of lithium chloride (LiCl) in rats has been measured at 526–840 mg/kg. The amount of lithium in the human body is about 7 mg; a dose of approximately 5 g LiCl can result in fatal poisoning [11]. On the other hand, lithium from drinking water is not expected to bioaccumulate and its environmental toxicity is low. The lithium requirement in humans is low; available experimental evidence appears to suggest that a provisional recommended daily allowance (RDA) for a 70 kg adult is 1 mg/day. This same evidence also states that the average daily intake of lithium of a 70 kg adult is around 0.65 to 3.1 mg/day from foods such as grains and vegetables [23,24].

Geochemically, lithium is a rare metal with average crustal abundances rarely exceeding 10–20 mg/kg. Lithium levels in granite (which makes up about two-thirds of Carlow’s bedrock geology) are higher at around 22 to 65 mg/kg. Lithium tends to be tightly bound in the crystal structure of the rock, therefore it alone does not pose an ecological problem [11,23,25,26,27,28]. Lithium does not appear in its pure form in nature due to its highly reactive properties. The metal occurs predominantly in the silicate matrices of pegmatites; some clay minerals such as Hectorite Na_0.3_(Mg,Li)_3_Si_4_O_10_(OH)_2_ [29]; geothermal brines; oilfield brines; and in solution in naturally occurring continental alkaline brine aquifers. The two major exploitable sources are pegmatite minerals such as spodumene and continental brine deposits. Brine deposits are easy to explore, fast to put into production and require less initial capital. Although lithium is easily extracted from brine sources, mineral deposits can contain much greater amounts of lithium. Lithium concentrations in subsurface brines range from 20 to 1500 mg/L [11,30], while mineral deposits may contain 530 to 55,100 mg/kg [31]. Hard rock mineral mining is a more involved process and usually takes the form of open pit mining depending on the depth at which the pegmatites occur. The processing of lithium-containing minerals involves crushing, wet grinding in a ball mill, sizing, gravity concentration followed by flotation using a fatty acid as a collector [11].

**Figure 1:**
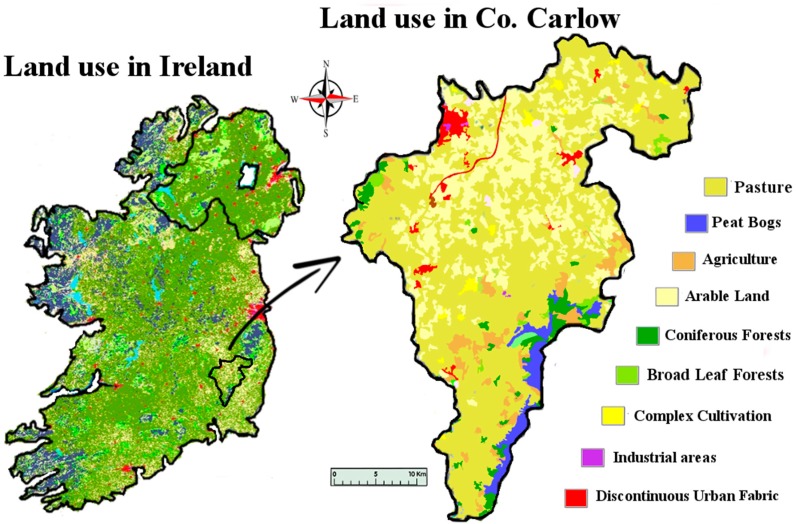
Map of Co. Carlow, showing land use. Image modified from [32,33].

The geology of the South East of Ireland is dominated by the Caledonian Leinster Batholith: a massive felsic intrusion of granite which occurred during the Caledonian orogeny about 419.2 ± 3.2 Mya. It is within this batholith that the lithium-rich pegmatites are found. First discovered in the late 1970s, the lithium deposit at that time was deemed to be of low economic value. Today however, the growth of the LIB market means that mining this once marginal deposit of lithium is a distinct possibility. These spodumene-bearing pegmatites represent one of the largest potential resources of lithium in Western Europe [31]. There are six known sites along the batholith associated with lithium bearing pegmatites: Aclare, Snowy Vale, Seskinnamadra, Stranakelly, Monaughrim and Moylisha. The largest discovered pegmatite deposit is at Aclare House, Myshall, County Carlow. There are no natural outcrops of the pegmatite deposit at Aclare; its presence was confirmed by drilling and the existence of pegmatite boulders in the area. Pegmatite deposits are extracted in one of two ways: either by open pit mining or underground mining, both of which may place a large load on the environment, i.e., mine tailings, the disposal and treatment of which is an important environmental issue for most mining projects. These tailings contain elements and compounds which are not naturally exposed to the ecological system and therefore have the potential to adversely affect the surrounding environment. The levels of lithium found in the waters of such tailings ponds have been measured at levels approaching 13 mg/L [11]. The post-disturbance pathway that these exposed metals take is likely to involve the natural waters of the locality. As well as being found in natural waters, metals originating from mining activities have been found in soil and plants. Farming is the dominant land use and industry in the study area (Figure 1). Carlow’s economy relies heavily on its agronomic sector, so any potential intrusion of lithium to the land is an important issue for the population. Lithium is readily absorbed by plants, and high levels of lithium have been shown to have harmful effects on plants. In some plants, lithium has been shown to stimulate growth. It has also been shown to cause growth depression in some citrus plants, causing drastic reductions in crop yields. Information on lithium hazard to other economic plants is limited [13,14,15,16,17,20,28].

It is important to understand the possible environmental impacts of extracting lithium in Ireland. The pegmatite deposits in Ireland exist as elongated tabular dykes. Mining of these pegmatites will probably take the form of open pit mining—one of the most common forms of strategic mineral mining. A good example of hard rock open pit pegmatite mining is the Talison Greenbushes and Wodgina mines in Western Australia which are the world’s leading producer of lithium from hard rock deposits. Open pit mining requires that pegmatite ores which have lain unexposed since deposition are brought to the surface for treatment. The ores are crushed and milled and then separated. The crushing creates dust which can contain radioactive elements, asbestos-like minerals and metallic particles which, absorbed into lung tissue, can cause problems such as pneumoconiosis and silicosis. Prolonged exposure to lithium dust can cause fluid to build up in the lungs, leading to pulmonary oedema. The separation process produces tailings (pulverized rock and liquids) from which toxic elements can leach into the bedrock and nearby water sources if not stored safely. The use of enormous amounts of water in the lithium extraction process is also an issue both for hard rock mining and brine deposits. Lithium brine mining operations in South America have seen several “water conflicts” arise due to the large amounts of water required by the lithium extraction industry [19]. The extraction of lithium in Ireland could potentially have harmful effects on the local environment through leaching, spills or air emissions. Any mining operation inevitably comes with a lengthy list of environmental concerns and potential issues. Public opinion and activism such as resistance to mining play an important role in deciding whether a deposit is recoverable. It is possible that public opposition may hinder any lithium mining operation in Ireland [34]. There is a need to measure baseline levels of lithium in the natural waters of the Carlow area prior to any mining operation. The purpose of this research was to establish those baseline levels of dissolved lithium and other metals in the natural waters of Carlow, namely the ground and surface water of the area; the rationale being that if mining were to take place in the region, the data produced here could be used as a benchmark to determine whether disturbed lithium, and by extension other metals we have analyzed, have been leached into the South East’s natural waters.

## 2. Materials and Methods

### 2.1. Sampling

**Figure 2:**
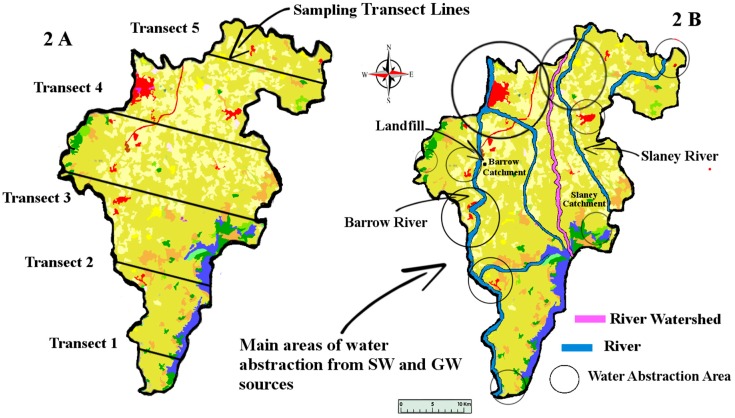
Map of Co. Carlow, showing locations of transect sampling lines. Image modified from [32,33]. SW: both surface; GW: groundwater.

Natural waters refer to both surface (SW) and groundwater (GW). This study aimed to sample county Carlow’s GW and SW. Carlow’s land area covers approximately 897 km^2^. GW and SW samples were taken along five transect sampling lines (Figure 2) crossing Carlow from East to West. These specific transect lines did not represent any significant geological features but were rather a numerical split of the area taking into account existing road access. This sampling method was chosen to sample the whole county in a cost-efficient way. This method also allowed us to address the question: has the lithium from the pegmatites located in the East/Central part of the county permeated towards the river Barrow in the West of the county.

**Figure 3:**
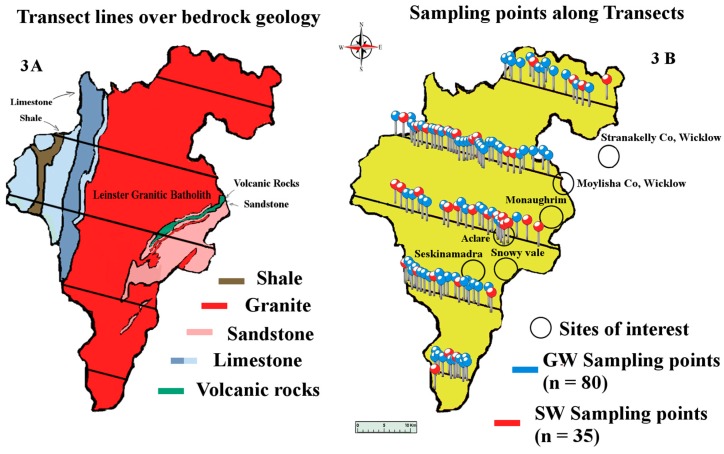
Map of Co. Carlow, showing bedrock geology, transect sampling lines, areas of interest and approximate sampling point locations. Image modified from [32,33].

The study was conducted over a period of seven months during 2015, from March to September. Water samples were collected every two months (four sampling events) from a total of 115 sampling sites, 80 of which were GW and 35 SW, giving a total of 460 water samples (Figure 3). GW samples were taken directly from household taps that were connected to private boreholes. SW samples were taken from streams and rivers along and adjacent to transect sampling lines. Water samples were collected in acid washed High Density Polyethylene (HDPE) sampling bottles. All bottles were acid washed with dilute HNO_3_, then filled with the dilute acid and allowed to sit for approximately one day. Bottles were then rinsed several times with deionized water and placed in sealable bags to prevent any contamination. Parts per billion measurements require rigorous cleaning and sampling methods; because of this, only ultra-pure analytical grade acids were used in this study. Samples collected in the field were filled to the brim leaving very little air space at the top of the bottle. Physico-chemical measurements (pH, Temperature and Conductivity) were taken in the field prior to filtering or acidification. Samples were filtered within 24 h of collection and then acidified. The samples were acidified to arrest any biological activity; dissolve any precipitates present; and discourage the adsorption of lithium onto the walls of the bottles. Trace metals are particularly prone to adsorption. Samples were stored at 4 °C ± 1 °C for the duration of the study.

### 2.2. Surface Water Sampling

SW samples (*n* = 140) were taken from rivers and streams where they intersected the sampling transect lines. Sample bottles were rinsed three times with the water being sampled. To avoid non-representative samples caused by surface films and the entrainment of river bottom deposits, a grab sampling method was used. Samples were taken from the center of rivers below the surface of the water while the sampler stood downstream of the sampling point. In some cases, (i.e., shallow streams), several small samples were amalgamated into a sufficiently large sample from which a final sample was taken.

### 2.3. Ground Water Sampling

GW samples (*n* = 320) were collected directly from home owners’ plumbing systems (i.e., domestic taps). Only sites which had their own GW well system installed were selected for the study. Care was also taken to avoid any water softening system or other treatment between the GW source and the tap. Prior to sampling from taps, the water was left to flow for two minutes before a sample was taken. When collecting GW samples, the container was rinsed three times with the water to be sampled before taking the final sample. Samples were also taken as close as possible to the source of the supply to minimize any potential influence from the plumbing system.

### 2.4. Sample Preparation

All water samples and blanks underwent the same preparatory procedures. Firstly, samples were concentrated by a factor of 10 by evaporation (200 mL samples were concentrated down to 20 mL before being analyzed). This ensured that the naturally low lithium levels in our samples would be measured at an order of magnitude greater than they appear naturally and therefore be more easily detected by our instrumentation. Samples were then filtered, using a slow filtration rate filter paper with particle retention at 2–3 µm to remove coarse and gelatinous precipitates from the solution. Further to this, samples were filtered using 0.45 µm pore size cellulose-based membrane syringe filters. These filters trap particles both on the surface of the filter and within the filter; consequently, the retention of small particles increased as the filter became more loaded. Samples were filtered avoiding excessive pressure. Algal cells are known to concentrate trace metals, so rupture of an algal cell could cause inaccurately high results; rupture may also introduce natural chelating agents into the water.

### 2.5. Reagents

Lithium Standard for AAS (certified reference material), TraceCERT^®^, 1000 mg/L Li in HNO_3_ was used to prepare all working standards. Potassium solution as an ionization suppressant, at 2000 mg/L was made up in double deionized water from Potassium hydroxide pellets for analysis (ACS,ISO,Reag. Ph Eur), EMSURE^®^, and acidified using HNO_3_. All working solutions and working standards were acidified using nitric acid (HNO_3_), ≥69.0%, for analysis EMSURE^®^ (ACS,Reag. Ph Eur) to the same percentage as collected samples (i.e., 1%). Glassware was thoroughly soaked in dilute HNO_3_ and rinsed several times with deionized water before use. No other metals in the analysis required an ionization suppressant. All reagents used were commercially available from Sigma Aldrich Ireland Ltd. Vale Road, Arklow, Wicklow, Ireland.

### 2.6. Analysis

All water samples were analyzed for lithium, as well as Cd, Cu, Fe, Mn, Ni, Pb and Zn using an Agilent (Tech 200 AA series) flame atomic absorption spectrometer (AAS) in an air-acetylene flame [35]. K and Na were analyzed using a Flame Emission Spectrophotometer (FES) Sherwood Model 410 Flame photometer. These instruments provided sufficient sensitivity, short analysis time and level of accuracy required for the study. Limit of Detection (LOD) for FES analysis was 0.13 mg/L while limit of quantification (LOQ) was 0.44 mg/L. LOD for all AAS analyses was 0.005 mg/L while LOQ was placed at 0.018 mg/L. The working range for lithium was 0 to 2 mg/L; working ranges were between 0 to 10 mg/L for all other metals. Samples were concentrated by a factor of 10 prior to any instrumental analysis, therefore a concentration factor of 10 was applied to all data before reporting the result. Processed samples were drawn at random before being read on the AAS to minimize procedural bias. After every 50 samples, the instrument was recalibrated using blank samples and working standards. Typical readings obtained from blank samples were 0.0001 to 0.0003 mg/L of lithium. Speciation of the lithium and the other metals analyzed in the water was not a factor in this study; only total dissolved metal content was measured.

### 2.7. Interferences

Alkali metals are very susceptible to ionization. In an air-acetylene flame, lithium ionization is appreciable. To control this ionization, all solutions were made to contain 2000 mg/L of the easily ionized potassium cation (ionization potential of 4.3 eV vs. 5.39 eV for lithium). At 2000 mg/L, minor changes in the potassium concentration had little or no effect on lithium’s absorbance readings.

### 2.8. Data Analysis

**Figure 4:**
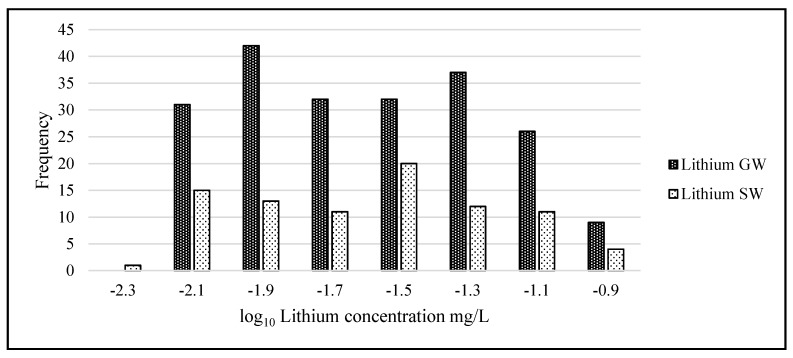
Histogram of SW and GW lithium concentration data. X-axis units are the logarithmic transformed mg/L data and Y-axis units are frequency. (*n* = 460 samples).

Statistical analysis was carried out using IBM’s statistical package SPSS (Statistical Package for the Social Sciences, version 23.0, IBM Corp, Armonk, NY, USA) and Microsoft Excel 2016. The lithium data were not normally distributed and when graphed showed considerable left skewness (GW skew = 3.89, kurtosis = 18.36; SW skew = 3.25, kurtosis = 12.11). To adjust the data to normality and allow the use of parametric tests, all data underwent a logarithmic transformation (Figure 4). When comparing lithium means among transects and geographical areas, a one-way ANOVA was used. Where significant differences were found, a post-hoc *t*-test was used to identify significant differences between sample means. A *p*-value of 0.05 was considered significant. Bonferroni corrections were used as appropriate.

## 3. Results

### 3.1. Statistical Analysis

**Figure 5:**
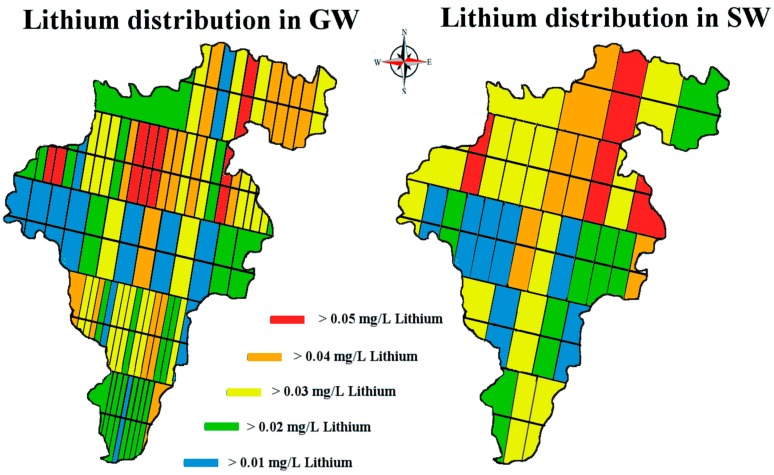
Map of Co. Carlow, showing lithium distribution levels in mg/L. Image modified from [32,33].

All water samples (*n* = 460) contained detectable amounts of lithium apart from 21 SW and 39 GW samples. In the case of GW sampling from people’s homes, we wanted to determine whether there was a difference when sampling from a pipe system that was unused overnight versus a system that had been used by a household all day, the null hypothesis being that there was no significant difference between the mean lithium levels measured in the morning and evening. One sampling point was selected from which 20 water samples were taken: 10 in the morning and 10 in the evening, then analyzed for lithium content. In total, 80 GW sites located along each of the five transect sampling lines were sampled four times giving a total of 320 observations. In total, 35 SW sites located along each of the five transect sampling lines were sampled four times from which a total of 140 observations were made. Surface water at $\bar{x}$ = 0.02, SD = 0.02 ranging from 0 to 0.091 mg/L and groundwater at $\bar{x}$ = 0.023, SD = 0.02 mg/L ranging from 0 to 0.097 mg/L (Figure 5).

A correlation analysis was carried out to assess whether there was any association between lithium and the physico-chemical variables (pH, Conductivity and Temperature). No significant correlations were observed with the variability explained, (*r*^2^) ranging from 0 to 6%. The mean lithium concentration in SW and GW appeared similar. A *t*-test (two-sample assuming equal variance) was carried out to test a null hypothesis that there was no significant difference between GW and SW mean lithium levels obtained from the entire data set, *n* = 460 observations (Ho: µgw − µsw = 0, vs. Alternative hypothesis: Ha: µgw − µsw ≠ 0). The test (*t* = 1.96, *df* = 458, *p*-value = 0.66) failed to reject the null hypothesis. There was no statistical difference between lithium levels in SW and GW.

### 3.2. Bimonthly Data

**Figure 6:**
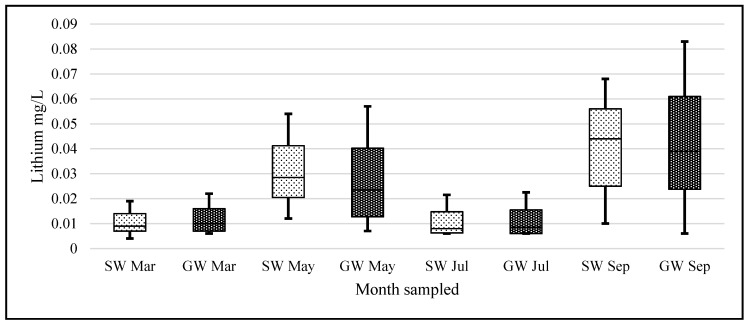
Boxplots of SW and GW lithium concentration data for each month. X-axis units represent months sampled and Y-axis units are mg/L lithium (*n* = 35 SW and *n* = 80 GW samples for each month).

Bimonthly sampling took place during 2015 with four different sampling events (March, May, July and September). One peculiar observation in the data can be seen in Figure 6. The variation in lithium levels in March and July appears similar as is the data in May and September. A one-way analysis of variance (ANOVA) conducted on our logarithmic transformed monthly lithium data—with a null hypothesis that there was no heterogeneity among months for lithium concentrations, (F (7, 288) = 34.67, *p* = 6.5 × 10^−35^)—showed a significant heterogeneity among the months.

Initially, we suspected that this relationship could be explained by procedural bias. Before any analysis began, all 460 SW and GW samples were mixed together. During the analysis, samples were selected randomly from this mix. Selecting samples using this method should have effectively negated the possibility of any procedural bias. Rainfall data for the area at the time of sampling did not correlate with the measured lithium concentrations; the above pattern was not present (March 53.5 mm. May 89.4 mm. July 79.4 mm. September 27.6 mm total rainfall in the sampling area) in the data [36]. To mitigate any diluting influence, sampling only took place two to three days after significant precipitation. We also considered that agricultural activities could have been responsible for the pattern. Land spreading of organic and chemical fertilizers is prohibited in Ireland during the times when the ground is likely to be frozen (September to January) under the European Union’s Nitrates Directive [37]. In the South-East, fertilizer application is prohibited from 15 September to 12 January [38]. In Ireland, farmers tend to apply fertilizer in the spring (February to April). The project’s sampling began in mid-March and ended in mid-September. At any time during our sampling, fertilizer spreading may have taken place. It is impossible to know when farmers spread fertilizer during our sampling, but we cannot ignore the possibility that agricultural activities may be responsible for the pattern. Ireland’s farmers rely heavily on N.P.K fertilizers (Nitrogen, Phosphorous and Potassium). There is also one landfill site located within Carlow (Powerstown Landfill and Recycling Centre) approximately 8 km South of Carlow town. This is almost exactly midway between transects 3 and 4 (Figure 2) and is thus unlikely to directly impinge on our measurements.

**Figure 7:**
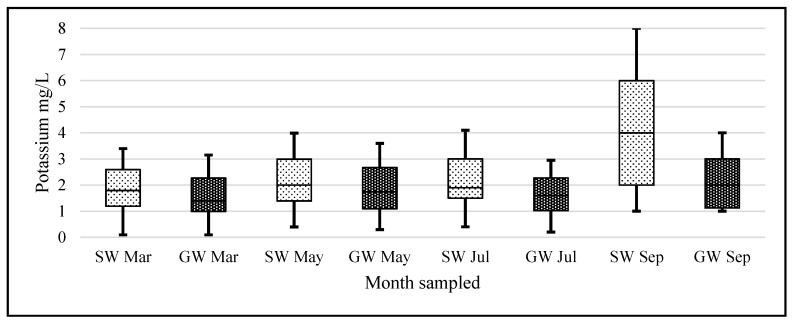
Boxplots of SW and GW potassium concentration data for each month. X-axis units represent months sampled and Y-axis units are mg/L potassium (*n* = 35 SW and *n* = 80 GW samples for each month).

**Figure 8:**
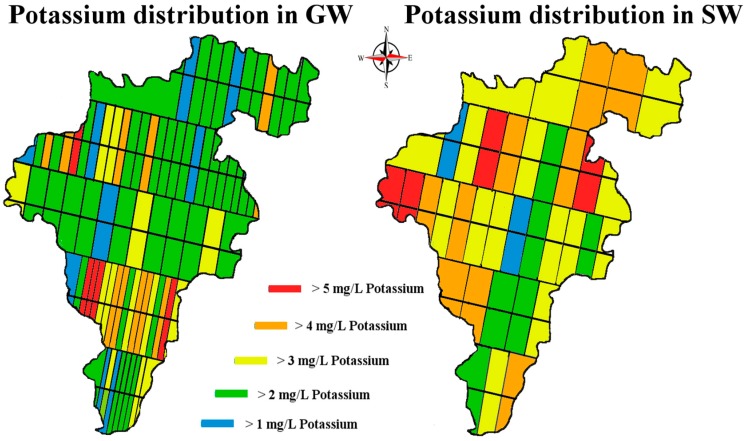
Map of Co. Carlow, showing potassium distribution levels in mg/L. Image modified from [32,33].

**Figure 9:**
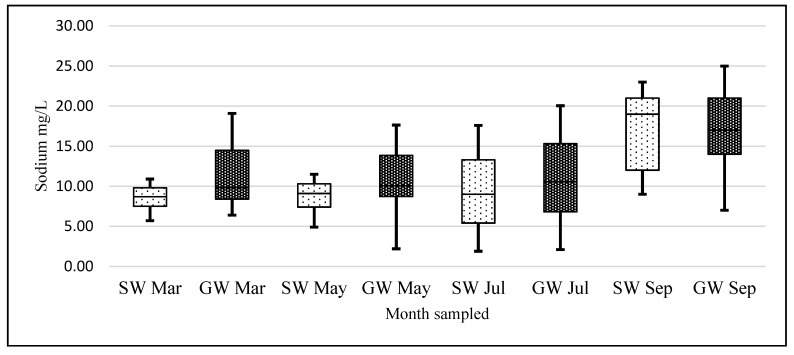
Boxplots of SW and GW sodium concentration data for each month. X-axis represents months sampled and Y-axis units are mg/L Sodium (*n* = 35 SW and *n* = 80 GW samples for each month).

**Figure 10:**
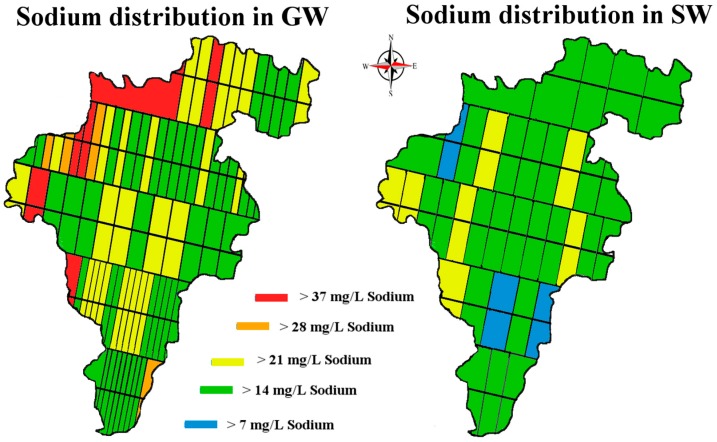
Map of Co. Carlow, showing sodium distribution levels in mg/L. Image modified from [32,33].

Data that we have collected for other alkali metals analyzed during the same time do not display the same pattern. For example, when a one-way analysis of variance (ANOVA) was conducted on our logarithmic transformed monthly potassium data, with a null hypothesis that there was no heterogeneity among months for potassium concentrations, the following was observed (F (6, 418) = 5.80, *p* = 8.08 × 10^−6^) rejecting the Ho. We do not observe the same pattern in our K data (Figure 7 and Figure 8). An ANOVA using the same hypothesis was conducted on the logarithmic transformed monthly sodium data, showing that the following (F (7, 452) = 6.17, *p* = 6.46 × 10^−7^) also rejected the Ho (Figure 9 and Figure 10). There is heterogeneity in the K and Na data but it is a different pattern than in the lithium data.

Nevertheless, the pattern of variation among months is different for the three elements, so we conclude that the heterogeneity is not due to procedural bias. We are currently unable to propose a mechanism to explain the observed pattern in the lithium data.

**Figure 11:**
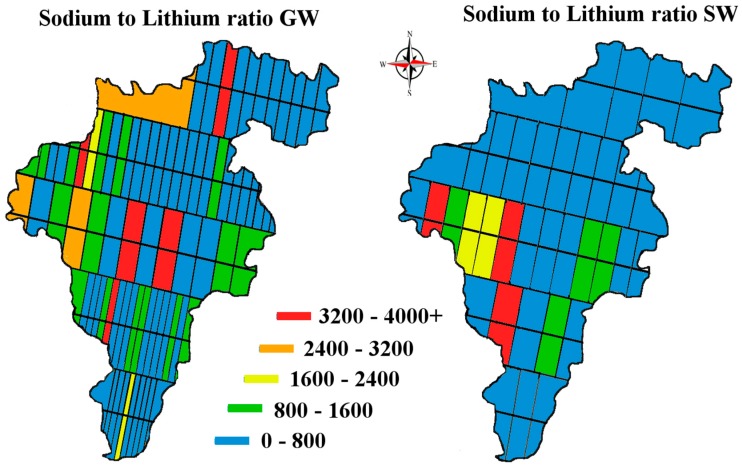
Map of Co. Carlow, showing the sodium to lithium distribution ratio in GW and SW, units mg/L. Image modified from [32,33].

Lithium enrichment is often expressed by the lithium to sodium ratio which is used as an indicator of residence time within an aquifer. The sodium to lithium ratio in the GW and SW of Carlow is shown in Figure 11 [39].

### 3.3. Transect Data

**Figure 12:**
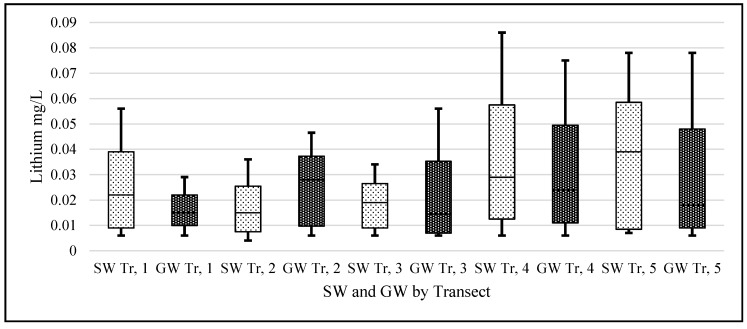
Boxplots of SW and GW lithium concentration data for each transect. X-axis represents the sampling transects and Y-axis units are mg/L Lithium (*n* = 35 SW and *n* = 80 GW samples for each month).

An ANOVA analysis was performed on the logarithmic transformed transect lithium data (Figure 12). The null hypothesis stated that there was no statistical difference between each transect’s mean lithium concentrations. In the case of both SW, (F (4, 82) = 1.88, *p* = 0.12) and GW (F (4, 204) = 2.44, *p* = 0.084), we failed to reject the null hypothesis when a Bonferroni correction for multiple testing was applied. This contrasts with the comparisons among months.

**Figure 13:**
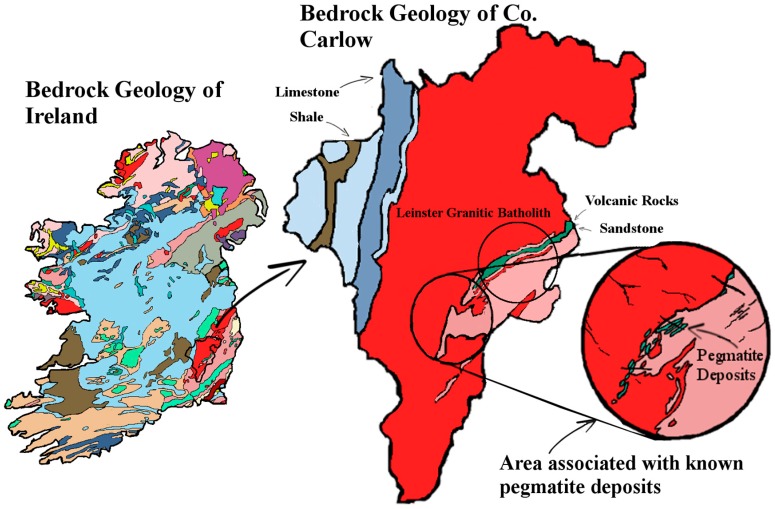
Bedrock geology map of Ireland and county Carlow showing general location of lithium bearing pegmatites. Image modified from [33].

The lithium bearing pegmatites are located along the Eastern border of the county; the Western border of the county is marked by the river Barrow valley. The river Barrow’s watershed boundary includes some of the areas of known pegmatite occurrences. One of the questions we sought to answer was whether the levels of lithium are higher in the East than in the West (Figure 13). This hypothesis was based on the highly mobile nature of the lithium ion and the weathering over time of the lithium bearing pegmatites. Dividing our data into West, Central and East data bins (the lithium bearing pegmatites being in the East of the county) and then conducting a *t*-test on the logarithmic transformed data—with the null hypothesis being that there was no difference between East and West lithium means—the following observations were made: for SW at (*t* = 1.98, *df* = 85, *p* = 0.24), we failed to reject the Ho and for GW at (*t* = 1.97, *df* = 207, *p* = 0.062), we also failed to reject the Ho.

### 3.4. Additional Metals

Along with lithium, potassium and sodium, seven other metals (Cd, Cu, Fe, Mn, Ni, Pb and Zn) were analyzed both in SW and GW. The results are listed in Table 1. The measured levels of each of these metals were within the safe range of threshold levels for drinking water set by the Irish Environmental Protection Agency (EPA) and the World Health Organization (WHO) and are of no immediate concern. Further, these levels did not exceed any maximum thresholds set for GW and SW by the Irish EPA [32,40]. The Irish EPA carries out routine water quality analyses all over Ireland, the data from which are publicly available [32]. These metals were chosen because they represent a common set of metals associated with water quality and to determine whether there were any significant correlations between lithium and the other metals. When a correlation analysis was carried out to assess this, no significant correlations were observed.

## 4. Discussion

The mean values obtained for lithium levels in county Carlow’s SW at $\bar{x}$ = 0.02, SD = 0.02 mg/L and GW at $\bar{x}$ = 0.02, SD = 0.02 mg/L are compatible with published figures for lithium concentrations in drinking waters; for example, drinking water in Texas 0.003 to 0.16 mg/L; in Oita, Japan 0.0007 to 0.059 mg/L; in England 0.001 to 0.021 mg/L; and in Portugal 0.001 to 0.19 mg/L [17,18,19,20]. It is worth noting that these figures are associated with studies investigating an association between lithium levels in drinking water and suicide rates in the local population. Some of these studies also claim a positive correlation among lithium levels, longevity and general mental health. The theory is that lithium in trace amounts enhances the connectivity among neurons and exposure over a lifetime enhances happiness [41]. However, data for Ireland, let alone Carlow, in relation to suicide are not currently available at sufficient geographical granularity to allow an investigation of potential associations between suicide rates and lithium levels in drinking water.

Lithium’s small ionic radius (0.68 Å) means that it has a high ionic potential and therefore has a strong affinity for water molecules. If the lithium from the known pegmatite sources is being slowly eroded and making its way down into the Barrow valley, we should have measured an elevated amount of lithium in our samples. Our data suggest that the lithium levels, although quite variable in the natural waters of Carlow, are within the range of normal ‘background’ levels reported in the literature and not elevated. The passage of water through the pegmatites is low. They are situated at an altitude above the level of the local water table and are unsaturated. The pegmatites also occur within a poor aquifer. The low concentrations found appear to be consistent with limited dissolution of the pegmatites.

When a one-sample *t*-test assuming unequal variances was carried out on our data—the null hypothesis being that the population mean was 0.010 mg/L; the alternative hypothesis being that our samples are greater than this level—the following was observed, (*t* = 1.96, *df* = 459, *p* = 3.1 × 10^−13^) rejecting the Ho. This should serve to focus some critical attention away from mean/median values to the range of levels for ‘lithium in natural waters’ when the literature and our data suggest variation through 3 orders of magnitude (0.0007 to 0.19 mg/L).

Finding bedrock outcrops of Ireland’s granitic batholith is quite rare. This is especially the case with the South East’s pegmatites. Neither recent nor historic prospecting have uncovered any cases of exposed pegmatite-bearing bedrock in the area. Pegmatites are very similar in composition to granite in that they both are susceptible to weathering. However, spodumene LiAl(SiO_3_)_2_ is an aluminosilicate mineral, thus the leaching from its lattice structure is very slow. This fact, the lack of exposed bedrock outcrops in the area and the fact that the pegmatite deposits are unsaturated may account for the low levels of lithium in our samples. Some of the compounds that lithium forms in nature such as its fluoride, carbonate and phosphate compounds also have very low solubility in water [42,43,44,45].

## 5. Conclusions

Lithium occurs at very low levels in water, and this elusiveness means that it is also an inherently difficult element to quantify. This research offers a snapshot of the lithium levels within the natural waters of the South East of Ireland. As with other studies, we have found low levels of dissolved lithium in natural waters but with significant heterogeneity through the year. This emphasizes the importance of repeated sampling to establish a true measure of lithium at a given site. From our work, we suggest that the following mean lithium values be used to establish baseline concentrations of lithium levels in the natural waters of the region: for surface water at $\bar{x}$ = 0.02, SD = 0.02 ranging from 0 to 0.091 mg/L and for groundwater at $\bar{x}$ = 0.023, SD = 0.02 mg/L ranging from 0 to 0.097 mg/L. These data establish a reference concentration for lithium in the natural waters of the area prior to any mining activity. The study may also be useful for other purposes: to assist in establishing a threshold value for lithium in natural waters, as a protocol of future baseline studies, to inform local water authorities and as a record for mining companies of the environmental conditions before ground is broken. Our analysis indicates that, undisturbed, the lithium-rich pegmatites of the Blackstairs have negligible effect on lithium concentration in local watershed.

## Figures and Tables

**Table 1:** Concentrations of analyzed metals in SW and GW given in mg/L (SD = Standard deviation, ND = Not Detected).

Metal	SW Mean	SW SD	GW Mean	GW SD
Cd	ND		ND	
Cu	0.08	0.43	0.12	0.28
Fe	0.1	0.21	0.05	0.19
K	2.7	1.7	2.2	2.1
Li	0.02	0.02	0.02	0.02
Mn	0.04	0.08	0.04	0.15
Na	11.1	5.2	15.5	14.2
Ni	0.01	0.01	0.01	0.04
Pb	0.04	0.05	0.04	0.07
Zn	0.08	0.19	0.14	0.23

SW: both surface; GW: groundwater.
